# Diversity and prevalence of gastrointestinal parasites in small ruminants in Franceville, Gabon

**DOI:** 10.1016/j.parepi.2025.e00465

**Published:** 2025-11-14

**Authors:** Krista Mapagha-Boundoukou, Larson Boundenga, Mohamed H. Mohamed-Djawad, Neil M. Longo-Pendy, Patrice Makouloutou-Nzassi, Mourad Ben Said, Gael Darren Maganga

**Affiliations:** a The Health Ecology Research Unit (URES) at Interdisciplinary Centre for Medical Research in Franceville (CIRMF), Franceville BP 769, Gabon; b The Regional Graduate School in Tropical Infectiology(EDR), Franceville BP 896, Gabon; c Anthropology Department at Durham University, South Road, Durham DH1 3LE, UK; d Laboratory of Evolutionary Biology Ecology and Ecosystem Management, Department of Animal Biology, Faculty of Science and Technology, Cheikh Anta Diop University, Dakar, Senegal BP 5005, Dakar, Senegal; e Department of Basic Sciences, Higher Institute of Biotechnology of Sidi Thabet, University of Manouba, Manouba 2010, Tunisia; f Laboratory of Microbiology, National School of Veterinary Medicine of Sidi Thabet, University of Manouba, Manouba 2010, Tunisia; g Unit of Emergence of Viral Diseases, at Interdisciplinary Centre for Medical Research in Franceville (CIRMF), Franceville BP 769, Gabon; h The National Higher Institute of Agronomy and Biotechnology (INSAB) is located at the University of Sciences and Technology of Masuku (USTM) in Franceville, Gabon; i Department of Biology and Animal Ecology, Research Institute of Tropical Ecology (IRET-CENAREST) BP 13354, Libreville, Gabon

**Keywords:** Gastrointestinal parasites, Sheep, Goats, Prevalence, Diversity, Risk factors, Gabon

## Abstract

Gastrointestinal parasites represent a major constraint on the health and productivity of small ruminants worldwide. In Gabon, and particularly in Franceville, data regarding parasite diversity, prevalence, and associated risk factors remain scarce. Understanding these aspects is crucial to develop effective, targeted control strategies and to safeguard animal health and food security. A cross-sectional study was conducted from August to October 2019, covering both dry and rainy seasons in Franceville, southeastern Gabon. A total of 120 fecal samples (113 sheep and 7 goats) were collected from twelve farms representing semi-intensive and extensive husbandry systems. Samples were analyzed using flotation and sedimentation coprological techniques to identify and quantify parasite eggs and oocysts. Host and environmental data, including age, sex, physiological status, farming system, and season, were recorded. Statistical analysis included prevalence estimation, Chi-square tests for association, and linear regression models with model selection based on Akaike's Information Criterion. The overall gastrointestinal parasite prevalence of 91.7 % was observed, consistent with regional African data. Ten parasite genera were identified, encompassing nematodes, cestodes, and protozoa. The most prevalent were *Oesophagostomum*/*Haemonchus* complex (64.6 %), *Eimeria* spp. (53.3 %), *Strongyloides* spp. (42.5 %), and *Trichostrongylus* spp. (38.0 %). Nematodes exhibited the highest mean infection intensity (∼1577 eggs per gram), followed by protozoa and cestodes. Risk factor analysis revealed that juveniles had higher prevalence of infestation, but lower parasite loads than adults, males carried higher burdens than females, and pregnant females had significantly elevated parasite loads (∼2.5 times higher). Extensive farming was associated with increased prevalence and burden, while seasonality influenced genus-specific occurrence. Limitations included small sample size for goats and absence of molecular diagnostics. This first study in Franceville (Gabon) demonstrates a high burden and diversity of gastrointestinal parasites in small ruminants, with key host and environmental factors influencing infection dynamics. The findings highlight the urgent need for genus-specific, integrated control measures adapted to local husbandry and seasonal patterns, especially targeting vulnerable groups like pregnant females. Future longitudinal studies employing molecular tools are recommended to refine parasite identification and optimize intervention strategies. Given the zoonotic potential of some parasites, a One Health approach is essential to improve animal health, public health, and food security in the region.

## 1 Introduction

Livestock production in Gabon plays a modest role in national food security and the economy, despite government initiatives to achieve food self-sufficiency (Galley, 2010; Sayouba, 2018). The country relies significantly on imported food, which constitutes 60–80 % of domestic consumption (Albert, 2005). In this context, small ruminants, particularly sheep and goats, are essential assets for rural households, providing meat, income and are not subject to any religious prohibitions (FAO et al., 2017). The sector is primarily characterized by two non-native breeds, namely the Djallonké sheep (*Ovis aries*) and the Fulani goat (*Capra hircus*). which are typically raised under extensive or semi-intensive management systems. Although not yet fully exploited, small ruminant farming represents a potential lever for improving food resilience at the community level in Gabon (Anim-Jnr et al., 2023).

Gastrointestinal parasites, including nematodes, cestodes, and protozoa, present a significant threat to the health and productivity of small ruminants globally. These pathogens lead to stunted growth, anemia, weight loss, and increased mortality, thereby reducing the economic value of livestock and perpetuating poverty among small-scale farmers (Coop and Kyriazakis, 2001; Lawrence et al., 2007). This is further exacerbated by the emergence of anthelmintic resistance, which reduces the efficacy of treatment (Leathwick and Besier, 2014). Furthermore, many gastrointestinal parasites are zoonotic, raising public health concerns in rural communities where human–animal interactions are prevalent (Feng and Xiao, 2011). In tropical environments such as Central Africa, climatic conditions (temperature, humidity, and rainfall) often favor the survival and transmission of parasitic stages in pastures, increasing the risk of infection (Harun et al., 2022; Short et al., 2017).

While studies have documented the prevalence of gastrointestinal parasites in ruminants across Africa (Ibrahim et al., 2014; Tumusiime et al., 2022), data specific to Gabon, particularly in semi-urban areas like Franceville, where traditional and modern farming practices converge, remains limited. Franceville's unique agro-ecological profile, combining peri-urban livestock management with traditional free-grazing systems, may influence parasite dynamics in ways that differ from rural or urban contexts. Understanding local parasite dynamics, risk factors, and host-parasite interactions is essential for developing targeted control measures that protect both animal and human health(Bautista-Garfias et al., 2022; Karimou et al., 2024). Such localized knowledge is also critical to informing veterinary extension services and guiding rational use of anthelmintics.

To address this knowledge gap, we conducted a cross-sectional study with the following objectives: (*i*) to identify the primary gastrointestinal parasite genera infecting sheep and goats in Franceville; (*ii*) to assess the impact of intrinsic (age, sex, and physiological status) and extrinsic (season and farming system) factors on parasite prevalence and burden; and (*iii*) to generate baseline data that supports integrated parasite management strategies in the region.

## 2 Materials and methods

### 2.1 Study area and period

The study was conducted from August to October 2019, covering both the dry and rainy seasons in Franceville, a city located in southeastern Gabon. This region lies within a humid tropical climate zone marked by distinct wet and dry periods, which significantly affect pasture availability and the epidemiology of gastrointestinal parasites in livestock. Average temperature ranges from 25 °C to 28 °C, and annual rainfall averages 2500 mm, creating favorable conditions for parasite development and transmission. Franceville was purposively selected for its diversity of livestock farming practices, ranging from traditional to semi-intensive systems, and due to the absence of baseline data on gastrointestinal parasitism in small ruminants. This city represents a semi-urban context where both modern and traditional rearing techniques coexist, making it an ideal site to study parasite diversity and transmission dynamics. Sampling was conducted over a three-month period corresponding to the transition between the rainy and dry seasons. Although relatively short, this period offers a representative snapshot of the climatic variability likely to influence parasite transmission in Franceville. However, sampling activities were partially constrained by logistical challenges and limited accessibility to certain sites.

### 2.2 Study population and sampling strategy

**Fig. 1:**
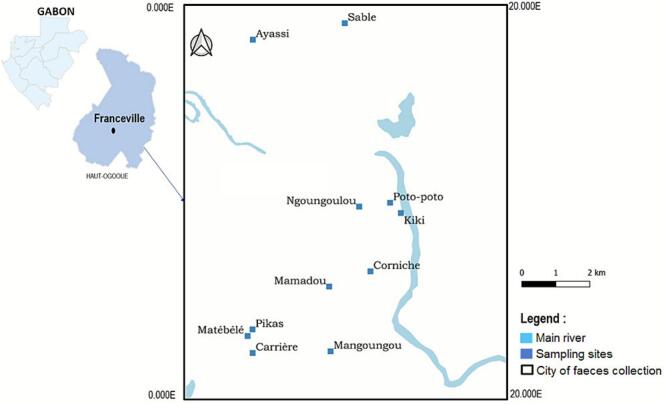
Sampling sites for sheep and goat fecal collection within the Franceville commune, Gabon.

A total of twelve farms located across eleven districts of the city were randomly selected from a provided list to ensure spatial representativeness and minimize selection bias (Fig. 1). The random selection process was conducted using a simple random sampling method, ensuring unbiased farm inclusion. Fecal sampling was carried out over three consecutive mornings each month from August to October 2019. Sampling mornings typically started between 6:30 AM and 11 AM, to ensure consistency and reduce environmental variation in sample quality. The study period coincided with the post-Ramadan period, during which many animals are traditionally slaughtered in family settings. Consequently, herd sizes were temporarily reduced, with breeding animals and juveniles comprising the majority of animals remaining on farms.

On each farm, approximately ten animals were randomly selected for sampling. The selection of animals used a random number table based on the daily herd census to avoid selection bias. Animals were classified as juveniles (≤2 years) or adults (>2 years), based on criteria established by (Emiru et al., 2013).

The physiological status of females was recorded, distinguishing pregnant, lactating, and non-pregnant individuals. Selection was limited to apparently healthy animals; individuals showing signs of severe illness (e.g., diarrhea, emaciation, lethargy) were excluded to avoid confounding effects on parasitological outcomes. This clinical screening was performed by qualified veterinarians through direct observation.

Sample size estimation was based on an assumed parasite prevalence (P) of 50 %, which represents the most conservative scenario to ensure adequate statistical power. Considering a finite population of approximately 1000 small ruminants in Franceville, the sample size (n) was calculated using the formula: n = Z^2^ × P×(1 − P)/d^2^, where: *Z* = 1.96 (standard normal deviate for 95 % confidence interval), *p-*value = 0.5 (expected prevalence), and *d* = 0.09 (desired precision or margin of error of 9 %). To account for possible sample loss or unusable data, the final targeted sample size was rounded up to 120 animals.

### 2.3 Husbandry systems and sanitary conditions

Two rearing systems were identified among the sampled farms: (*i*) Semi-intensive system, characterized by animals housed in enclosures overnight and allowed access to pasture & system, involving minimal human intervention where animals roam freely in surrounding neighborhoods and forage naturally (Delanoue and Roguet, 2013). Approximately six farms practiced the semi-intensive system, while six others followed an extensive management approach. Hygienic conditions were generally poor across study sites, with rudimentary animal housing and limited waste management. For instance, an aborted fetus was observed discarded on the ground in one farm, illustrating a lack of veterinary oversight and the potential risks to both animal and public health. Such conditions may facilitate parasite transmission and complicate control efforts in the region. These observations will be discussed further in the Discussion section. Fecal samples were collected manually from the rectum using gloved hands and placed into clean, labeled containers. Samples were transported in cool boxes to the laboratory and processed within 24 h of collection to maintain sample integrity.

### 2.4 Sample handling and laboratory processing

Fecal samples were collected either immediately after spontaneous defecation or directly from the rectum using gloved fingers. Approximately 3 g of feces were placed into sterile 45 mL Falcon tubes and stored at 4 °C until analysis (Soulsby, 1982). To optimize sample quality, all processing was completed within 48 h of collection to prevent larval development and degradation of parasitic stages.

Upon arrival at the laboratory, samples were first homogenized and macroscopically inspected for adult parasites or proglottids. Parasitological analyses were then performed using both flotation and sedimentation techniques, following the protocols described by Dryden et al. (2005) and Lester and Matthews (2014), with minor modifications. Specifically, bromothymol blue staining was omitted to avoid interference with the optical contrast and enhance the clarity of egg and oocyst visualization. The flotation method was used primarily for detecting nematode and cestode eggs and coccidian oocysts, while the sedimentation technique allowed for improved identification of heavier eggs such as those of trematodes.

In the absence of larval culture, each morphotype was characterized by its mean egg length and width, standard deviation and number of blastomeres observed, in order to refine the classification. These morphometric parameters are summarized in Supplementary Table S1, in which morphotypes 1, 2 and 3 correspond morphologically to the genera Haemonchus, Oesophagostomum and Bunostomum, respectively. This approach improves the reliability of genus-level identification in coprological examinations as previously described (Prijma et al., 2023; Soulsby, 1982).

Quantification of infection intensity was conducted by calculating eggs per gram (EPG) of feces using the following formula: EPG = n_1_ + n_2_/2 × 100, where *n₁* and *n₂* represent the egg counts in two separate microscope fields. For standardization, counting was performed using the McMaster technique with calibrated counting chambers. The intensity of infection was classified based on the scale defined by Alowanou et al. (2021), allowing for intra- and inter-genera comparisons. The classification thresholds were as follows: light infection (< 200 EPG), moderate infection (200–500 EPG), and heavy infection (> 500 EPG).

### 2.5 Statistical analysis

Statistical analyses were conducted using R software (version 3.6.3, R Core Team, 2020). The overall prevalence of gastrointestinal parasites was calculated as the proportion of positive samples relative to the total number of animals examined, expressed as a percentage. Exact binomial 95 % confidence intervals were also computed to assess the precision of prevalence estimates. Although this approach provides a reliable approximation of apparent prevalence, its accuracy may have been influenced by factors such as sample representativeness and the degree of randomization in farm and animal selection. A set of explanatory variables, selected based on previous findings and biological relevance, included age group (juvenile [≤2 years] or adult [>2 years]), sex (male or female), species (sheep or goat), season (dry or rainy), physiological status (pregnant, lactating or non-pregnant/non-lactating), and rearing system (extensive or semi-intensive). Differences in parasite prevalence among these categories were assessed using Pearson's Chi-square test (χ^2^), and Fisher's exact test was applied when expected frequencies were too low. A *p*-value below 0.05 was considered statistically significant. For continuous outcomes such as parasite burden, measured in eggs per gram (EPG) of feces, linear regression models were used to evaluate associations with the aforementioned variables. EPG data were visually examined for normality and homoscedasticity, and log-transformation (e.g., log₁₀ (EPG + 1)) was applied where necessary to satisfy regression model assumptions. To identify the most parsimonious and best-performing model, all possible combinations of explanatory variables were ranked using the dredge () function from the *MuMIn* package, based on Akaike's Information Criterion (AIC). The model with the lowest AIC (ΔAIC = 0) was retained as the final model for interpretation, ensuring a balance between fit and simplicity.

## 3 Results

### 3.1 Demographic characteristics of the study population

A total of 120 small ruminants were included in the study, comprising 113 sheep and 7 goats, belonging to the Djallonké (109) and Fulani (11) breeds. These animals were sampled across two farming systems, with 59 individuals (49.2 %) originating from semi-intensive farms and 61 (50.8 %) from extensive farms. The study population consisted of 80 females (66.7 %) and 40 males (33.3 %). Among goats, there were 3 females (42.9 %) for every 4 males (57.1 %); among sheep, there were 77 (68.1 %) females for every 36 males (31.9 %). In terms of age distribution, 34 animals (28.3 %) were identified as juveniles (≤2 years), while the majority, 86 animals (71.7 %), were adults with an average age of 43 months. Regarding physiological status, among the female animals, 29 (36.3 %) were pregnant and 51 (63.8 %) were non-pregnant. Seasonal distribution of the sampling showed that 90 animals (75 %) were collected during the dry season and 30 (25 %) during the rainy season, corresponding to the months of august for dry, October for rainy season. Overall, the sample was relatively balanced across production systems and age groups, but skewed toward sheep and female animals. All demographic data are summarized in Table 1, which presents the distribution of animals by species, sex, age group, physiological status, farming system, and season of collection.

**Table 1:** Prevalence rates of gastrointestinal parasites according to analyzed factors.

Factors	Classes	Total	Positive	Prevalence rate (% ± C.I.^1^)	*p*-value
Species	Sheep	113	104	92.0 ± 0.049	0.558
	Goat	7	6	85.7 ± 0.258	
Type of farm	Semi-intensive	59	56	94.9 ± 0.056	0.207
	Extensive	61	54	88.5 ± 0.080	
Gender	Female	80	72	90.0 ± 0.066	0.352
	Male	40	38	95.0 ± 0.066	
Age	Young (<2 years)	34	32	94.1 ± 0.078	0.543
	Adult (≥ 2 years)	86	78	90.7 ± 0.060	
Physiological status	Non-pregnant	51	43	84.3 ± 0.099	0.045^⁎^
	Pregnant	29	29	100	
Season	Dry	90	66	73.3 ± 0.054	0.434
	Rain	30	27	90.0 ± 0.107	
Total		120	110	91.7 ± 0.049	–

Abbreviations: ^1^:C.I.: 95 % confidence interval, ^⁎^: Statistically significant, *p*-value <0.05.

### 3.2 Overall prevalence and parasite diversity

Microscopic examination of fecal samples revealed a high overall prevalence of gastrointestinal parasitism, affecting 91.7 % of the sampled animals (110 out of 120). Prevalence was slightly higher in sheep (92.0 %) compared to goats (85.7 %), although this difference was not statistically significant (*p-*valu*e* > 0.05). A total of nine genera of gastrointestinal parasites were identified, representing seven helminthes, including five group of nematodes (*Strongyloides* spp., *Trichuris* spp.*,* strongyles 1, strongyles 2 et strongyles 3) and one cestode (*Moniezia* spp) as well as three protozoan genera (*Eimeria* spp., *Balantioides* spp., and *Entamoeba* spp.).

**Fig. 2:**
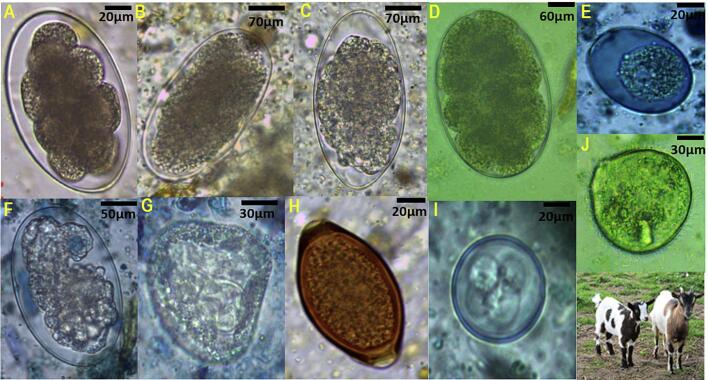
Microscopic observations of gastrointestinal parasite eggs identified in small ruminants (n = 120) from Franceville, Gabon. Legend: A, Strongyles 3; B, Stronyles 1; C, *Trichostrongylus* sp.; D, Strongles 2; E oocyst eggs, F, *Strongyloides* sp.; G *Moniezia* sp.; H, *Trichuris* sp.; I, *Eimeria* spp.; J, *Balantidiois* sp.; and K, The hosts example. Were analyzed using flotation (Willis method) and sedimentation techniques under an optical microscope at 400× magnification. Images were captured to illustrate representative morphotypes of the intestinal eggs parasites identified.

**Fig. 3:**
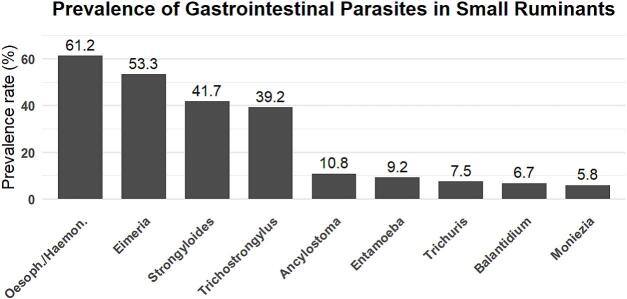
Prevalence rates of all intestinal parasite eggs identified in small ruminants (*n* = 120) from Franceville, Gabon.

The most prevalent genera were *Eimeria* spp. (53.3 %), *Strongyloides* spp. (42.5 %), *Trichostrongylus* spp. (38.0 %) strongyles 1(33.3 %) and strongyles 2 (30.83 %). Less frequently detected parasites included *Trichuris* spp., *Balantioides* spp., *Entamoeba* spp., *Moniezia* spp. and strongyles 3, each with low detection rates across the study population. Detailed prevalence figures per genus and species are presented in Table 2 and Fig. 3, while Fig. 2 illustrates representative microscopic images of the most common egg and oocyst morphotypes observed.

**Table 2:** Prevalence rates of gastrointestinal parasites according to various risk factors.

Parasite group Genus	EPG./OPG	Means /sd	Types of	Animal species		Type of farm		Gender		Age		Physiological status		Season	
	means		infection	Sheep	Goat	Extensive	Semi-intensive	Female	Male	Young	Adult	Non-pregnant	Pregnant	Dry	Rain
Nematodes	1780	734.58 ± 826.87	High												
*Strongyloides*	308			42.48	28.6	42.4	41.0	45.0	35.0	44.11	40.7	54.2	31.0	46.7	26.7
*Trichostrongylus*	267			38.05	57.1	35.6	39.3	33.8	50.0	41.18	38.4	31.3	41.4	34.4	46.7
*Trichuris*	395			7.96	0	3.4	13.1	6.3	10.0	14.71	4.7	6.3	10.3	10	0
Strongyles 1	153			32.1	6	4.0.6	14.3	7.8	13.5	17.25	6.5	9.2	0	5.6	4.2
Strongyles 2	274			30.83	57.1	59.3	68.9	57.5	77.5	61.76	65.1	50.0	72.4	72.2	40.0
Strongyles 3	180			11.5	0	5.1	16.4	10.0	12.5	8.82	11.6	8.3	13.8	4.4	3.3
Cestodes	614	20.83 ± 226.23	Sligh												
*Moniezi*a	614			4.42	28.6	6.8	8.2	2.5	12.5	8.82	4.7	2.1	3.4	3.3	13.3
Protozoa	822	355.18 ± 864.75	Moderate												
*Balantioides*	144			7.08	0	6.8	3.3	7.5	5.0	5.9	7.0	6.3	10.3	3.3	16.7
*Entamoeba*	…			8.84	14.3	11.9	6.6	8.8	10.0	5.9	10.5	4.2	6.9	6.7	6.7
*Eimeria*	678			53.1	57.1	71.2	39.3	60.0	40.0	50.0	54.7	56.3	65.5	46.7	73. 3

Abbreviations: EPG: Eggs Per Gram.

**Fig. 4:**
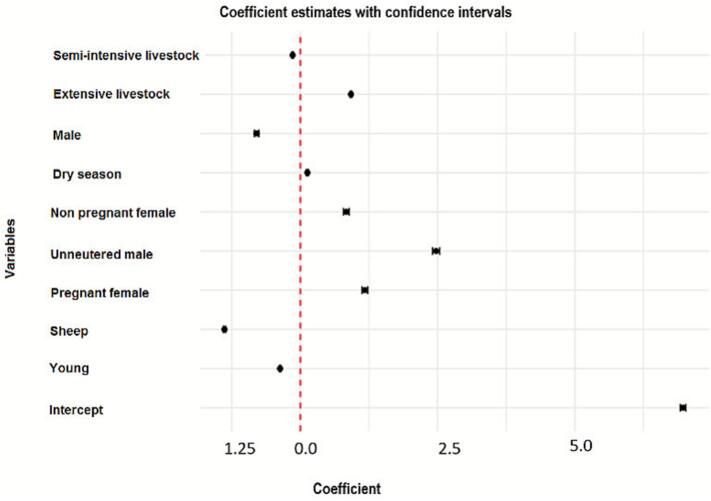
Effect of parasite load on host and environmental factors.

### 3.3 Intensity of infection

The intensity of gastrointestinal parasite infections, expressed as eggs per gram (EPG) for helminths and oocysts per gram (OPG) for protozoa, showed notable variation among the different parasite groups. Nematodes exhibited the highest mean infection intensity with 734.58 ± 826.87 EPG, followed by protozoan parasites with an average of 355.18 ± 864.75 OPG, and cestodes with the lowest burden at 20.83 ± 226.23 EPG. Based on the classification thresholds defined by (Alowanou et al., 2021), nematode and protozoan infections were categorized as to high intensity to moderate, with EPG/OPG values >200, whereas cestode infections fell into the mild to light intensity range (Table 2). Statistical analysis confirmed significant differences in mean infection intensities between parasite groups (ANOVA, F = 8.47, *p-*value =0.0003 < 0.05), with post-hoc multiple comparisons using Tukey's test (α = 0.05) revealed significant differences among the parasite groups. Nematode exhibited a significantly higher mean load compared to protozoa (mean difference = +379.40; 95 % CI [98.10–660.70]; *p-*value = 0.004) and cestode (+713.75; 95 % CI [432.45–995.05]; *p-*value <0.001). Furthermore, protozoa showed a significantly greater infection intensity than cestode (+334.35; 95 % CI [53.05–615.65]; *p-*value = 0.017). Overall, these findings indicate a clear hierarchy of parasite burdens: Nematode > Protozoa > Cestode (*p-*value <0.05 for all pairwise comparisons).

### 3.4 Parasite prevalence by host and environmental factors

High prevalence rates of gastrointestinal parasite infection were observed across all evaluated subgroups (Table 1). Overall, infection was slightly more frequent in males 95 %, compared to females 90 %. Similarly, juveniles (≤ 2 years of age) exhibited a higher prevalence 94.1 %, than adults (> 2 years), who showed a prevalence of 90.7 %. Seasonal variation impacted infection rates, with prevalence significantly higher during the rainy season 90 %, compared to the dry season 73.3 %, potentially reflecting environmental conditions favorable to parasite transmission. Regarding rearing systems, animals raised under extensive management showed significantly increased infection rates 94.9 % compared to those in semi-intensive systems 88.5 %. Physiological status was significantly associated with infection (*p-*value = 0.045), with all pregnant females 100 % testing positive, in contrast to 84.3 % infection prevalence among non-pregnant females.

### 3.5 Impact of factors on parasite occurrence

Generalized linear models (GLMs), assuming a binomial distribution for parasite presence/absence data, were employed to assess the influence of host and environmental factors on gastrointestinal parasite prevalence. While physiological status showed a significant association with overall parasite occurrence in univariate analyses, it did not remain a significant predictor in the multivariate model, suggesting confounding or interaction effects with other variables. Distinct host and environmental factors exerted genus-specific effects on parasite prevalence.

The analysis of risk factors revealed that the farming system, host sex, season, and age were significant determinants of parasitic prevalence. The farming system was a significant determinant for strongyle 3, *Balantioides* spp., strongyles 1, strongyles 2 and *Trichuris* spp., with semi-intensive farms showing significantly higher prevalence rates than extensive farms. In contrast, the prevalence of *Eimeria* spp. was not significantly associated with the farming system.

Host sex significantly influenced the prevalence of certain parasites. *Entamoeba* spp. and *Trichostrongylus* spp. were significantly more prevalent in males, with infection rates 71 % (*p* < 0.05) and 46 % (*p-*value <0.05) higher than in females, respectively. Notably, *Moniezia* spp. infection was observed exclusively in males, with no cases detected in females.

Seasonal variations were a significant predictor of prevalence. A consistently positive effect was found for *Balantioides* spp. (95 % CI: 0.87–1.78; *p*-value <0.001), *Moniezia* spp. (95 % CI: 1.41–2.69; *p-*value <0.001), *Strongyloides* spp. (95 % CI: 0.52–1.36; *p-*value = 0.002), and the strongyles 2 (95 % CI: 0.39–1.23; *p-*value = 0.008), indicating a markedly increased risk of infection during the wet season, likely due to enhanced survival of infective larval stages in the environment. Finally, age was a key determinant for *Trichuris* spp. (95 % CI: 1.89–3.53; *p-*value <0.001). Juveniles exhibited a markedly higher prevalence (28.6 %) compared to adults (4.3 %), corresponding to a relative risk of 6.7 (95 % CI: 2.1–21.3; *p-*value <0.001) (Table 3).

**Table 3:** Effect of host and environmental factors on the prevalence of different parasites.

Parasites	Age	Species	Physiological statut	Season	Gender	Type of farm	AICc		Δ
*Balantidiois* spp.	−	+	−	+	−	+	55.5		0
*Eimeria* spp.	−	−	−	−	−	+	146.6		0
*Entamoeba* spp.	−	−	−	−	+	−	73.0		0
*Moneizia* spp.	−	−	−	+	+	−	49.8		0
*Strongyloides* spp.	−	−	−	+	−	−	163.9		0
*Trichostrongylus* spp.	−	−	−	−	+	−	161.9		0
*Trichuris* spp.	+	−	−	−	−	+	54.0		0
Strongyles 1		−				+	131.2		0
Strongyles 2	−	−	−	+	−	+	132.0		0
Strongyles 3	−	−	−	−	−	+	81.8		0

Abreviations: +: Significant effect, −: Non-significant effect, AICc: Akaike Information Criterion, Δ: Delta AICc.

### 3.6 Factors affecting parasite load in animals

Regression analysis identified several host- and environment-related factors that significantly influenced the parasitic load in small ruminants (Fig. 4). Adult animals exhibited a significantly lower parasitic load than juveniles (RR = 0.76; 95 % CI: 0.65–0.89; *p-*value = 0.001), suggesting the potential development of acquired immunity with age. Conversely, females had a parasitic load 1.15 times higher than males (RR = 1.15; 95 % CI: 1.02–1.30; *p-*value = 0.025), reflecting potential differences linked to sex-based dimorphism in immune response or physiological factors.

Although goats presented a slightly higher parasitic load than sheep (RR = 1.10; 95 % CI: 0.95–1.28; *p-*value = 0.210), the small sample size of goats (*n* = 7) limited the statistical power of this comparison. As expected, pregnant females carried a parasitic load 2.5 times higher than non-pregnant females (RR = 2.5; 95 % CI: 2.1–3.0; *p-*value <0.001), which aligns with the well-documented immunosuppressive effects of gestation.

Contrary to initial hypotheses, the dry season did not have a significant effect on parasitic load (RR = 1.08; 95 % CI: 0.93–1.25; *p-*value = 0.320) compared to the wet season. Finally, animals raised in extensive farming systems had significantly higher parasitic loads than those in semi-intensive systems (RR = 1.40; 95 % CI: 1.20–1.63; *p-*value <0.001), underscoring the impact of management practices on infection intensity (Fig. 4).

## 4 Discussion

This study provides the first robust epidemiological investigation of gastrointestinal parasite diversity and prevalence in small ruminants in Franceville, Gabon, highlighting a strikingly high infection rate of 91.7 %. Such levels threaten both animal productivity and rural livelihoods, with direct consequences for food security. While similar prevalences are reported across the continent, Rwanda (100 %; (Galley, 2010; Sayouba, 2018)), Cameroon (93.2 %; (Mbuh et al., 2008)), Nigeria (87.5 %; (Eke et al., 2019)), this work formally documents the situation for Gabon and underscores the urgent need for regional control strategies. The high prevalence likely results from a combination of conducive climatic conditions (persistent humidity and high temperatures) and predominantly extensive husbandry practices, which together facilitate parasite transmission (Anagonou et al., 2020; Van Dijk and Morgan, 2012). Although the three-month sampling window (August to October 2019) captured both dry and rainy seasons, this temporal restriction limits detailed inference on seasonal parasite dynamics. Extending future surveillance to a full annual cycle would better inform intervention timing (Hoste et al., 2008; Leathwick and Besier, 2014).

The spectrum of identified parasites reveals a clear epidemiological pattern. The pronounced prevalence of *Eimeria* spp. (protozoa) and *Strongyloides* spp. (nematodes) points to environmental conditions and management practices that favor the direct, fecal-oral transmission cycle. This is characteristic of environments with high humidity and suboptimal sanitation, where oocysts and larvae can readily persist and develop to infective stages (Ali et al., 2025; Tiele et al., 2023). This pattern was further reinforced by the substantial recovery of trichostrongylid nematodes, particularly *Trichostrongylus* and Strongyle 1 (*Haemonchus* spp). The free-living stages of these genera are known to thrive in the warm, moist conditions of tropical pastures, explaining their significant contribution to the overall parasitic load(Khadijah et al., 2013). In contrast, the comparatively low prevalence of cestodes like *Moniezia* spp. suggests a different dynamic. Their life cycle, which requires an intermediate host (oribate mites), presents an additional ecological constraint. This lower frequency could be attributed to limited host-parasite contact, potentially due to fluctuating mite populations, specific pasture ecologies unfavorable to the intermediate host, or seasonal variations not captured in this cross-sectional study(Ismayilov et al., 2014; Mohamed et al., 2023).

The detection of nine parasite genera, comprising five nematodes, two cestodes, and four protozoa, reflects a high level of diversity on par with endemic regions like Egypt (13 genera; Abbas and Hildreth, 2022) and Ethiopia (11 genera; Ibrahim et al., 2014). This parasite spectrum evidences the complexity of host-parasite interactions in the humid tropics and their potential cumulative impact on animal health and productivity. Nonetheless, the low number of goats sampled (*n* = 7) prevents firm interspecies comparisons; methodological recommendations point to at least 30 animals per species for robust analysis (Win et al., 2020). Consequently, future work should ensure more balanced species representation. While coprological methods provided valuable morphological identification, their limitations, especially for morphologically similar strongyles are clear (Taylor et al., 2015). The adoption of molecular diagnostic tools (e.g., ITS2-targeted PCR, qPCR) in subsequent studies will increase identification accuracy and support more targeted and effective parasite management programs. It is worth noting that several Eimeria oocyst morphotypes of varying sizes were observed, suggesting the possible presence of multiple species. However, due to the absence of sporulation or molecular analyses, we deliberately restricted our identification to the genus level to avoid taxonomic bias. Future studies incorporating sporulation or molecular characterization are needed to accurately determine the Eimeria species circulating in small ruminants in Gabon (Liu et al., 2024).

Nematodes exhibited the highest parasite loads in this study, reaching up to 1780 eggs per gram (EPG), while infections with *Eimeria* and *Moniezia* spp. exceeded 600 eggs per gram, indicating moderate to severe infestations (Agride Agride, 2012). Such elevated parasite burdens likely reflect the environmental resilience of these parasites, which thrive particularly in farming systems characterized by suboptimal sanitation and hygiene practices (Houert, 2018). These high infection intensities are of considerable clinical relevance as they can directly impact animal health and productivity. Heavy nematode burdens may cause anemia, reduced weight gain, and increased mortality. Likewise, *Eimeria* spp. infections are known to cause enteric diseases such as coccidiosis, especially in young or immunocompromised animals, potentially leading to severe diarrhea and poor growth performance.

Our univariate analyses revealed a significant association between pregnancy and higher parasite prevalence (*p*-value = 0.045). However, this relationship did not remain significant in multivariate models, possibly due to confounding effects of other factors such as age or husbandry system. Nonetheless, pregnant ewes exhibited parasite loads 2.5 times higher than their non-pregnant counterparts, an observation consistent with studies highlighting pregnancy-induced immune stress as a risk factor for increased susceptibility to infections (Islam et al., 2017; Singh et al., 2017). These findings suggest that physiological stress during pregnancy compromises the host's immune defenses, facilitating higher parasite burdens, which may negatively affect both maternal health and offspring viability. Consequently, the management of parasitic infections in pregnant females should be prioritized within integrated parasite control programs. These high parasite intensities underscore the need for targeted interventions emphasizing improved sanitation and tailored treatment strategies, particularly in extensive farming contexts where reinfection pressure is intensified. Future research should aim at longitudinal monitoring of parasite burdens relative to physiological stages to optimize control measures and reduce economic losses in small ruminant production.

Age and sex significantly influenced both parasite prevalence and burden in the studied small ruminants. Juveniles exhibited a higher prevalence of infestation but carried lower average parasite loads compared to adults. This pattern reflects a common epidemiological dynamic where younger animals with naive immune systems are more frequently infected but develop protective immunity as they age, resulting in a reduced ability of parasites to establish and persist, i.e., lower fecal egg counts, despite continued exposure (Tembely et al., 1997; Zvinorova et al., 2016). This acquisition of immunity with age is a well-documented phenomenon in gastrointestinal nematode infections (Roeber et al., 2013). In contrast, males harbored higher parasite loads than females, possibly explained by behavioral differences such as increased roaming or aggression, and hormonal influences including immunosuppressive effects of androgens (Klein, 2004). However, females exhibited a slight overall predominance in prevalence, which may be linked to physiological states, particularly during gestation and lactation, where pregnancy-induced immunosuppression increases susceptibility and parasite establishment (Singh et al., 2017; Islam et al., 2017). This immunological depression during the periparturient period is well documented in ruminants (Houdijk, 2008; Houdijk et al., 2001) and explains the significantly higher parasite burdens observed in pregnant females of this study.

Husbandry practices exerted a critical role in shaping parasite infection patterns. Animals raised under extensive systems demonstrated significantly higher prevalence and parasite loads compared to those in semi-intensive systems, highlighting the detrimental impact of limited health control, low hygiene standards, and free grazing practices favorable to parasite transmission (Chikweto et al., 2018; Maouche, 2021). Extensive systems often involve unrestricted access to contaminated pastures and lack of routine anthelmintic treatment, which perpetuates infective stages in the environment (Gouveia et al., 2013). In contrast, semi-intensive systems, with partial confinement and supplementary feeding, potentially reduce exposure risk and improve overall animal resilience. Improving pastoral management through strategies such as reducing stocking density, implementing rotational grazing, and enhancing hygiene has proven effective in mitigating parasitic burdens (Hoste et al., 2008; Waller, 2006). These measures limit pasture contamination and break parasite life cycles, thereby reducing infection pressure (Prasad et al., 2019; Rapiya et al., 2019).

Seasonality also influenced parasite prevalence and intensity. Although the study covered both dry and rainy seasons, the rainy season was associated with increased gastrointestinal parasite prevalence and load, possibly due to the favorable climatic conditions (e.g., humidity, temperature) that enhance the survival and development of free-living parasite stages on pastures (Castro et al., 2023; Karimou et al., 2024). This seasonal pattern aligns with findings from similar tropical settings, where parasite transmission peaks coincide with periods of high rainfall and vegetative growth (Nwosu et al., 2007). Effective control programs should therefore be tailored to these seasonal dynamics to optimize timing and efficacy. Moreover, detection of zoonotic genera, notably *Strongyloides, Entamoeba,* and *Balantioides*, signifies a substantial cross-species transmission risk. Their presence is a direct indicator of environmental contamination and inadequate sanitation, creating a conduit for parasite exchange between animal and human populations. These findings unequivocally underscore the necessity of a One Health framework, which integrates robust veterinary surveillance, stringent farm hygiene protocols, and public health education to mitigate zoonotic threats in shared ecosystems(Esteban-Sánchez et al., 2024; Ottino et al., 2020). In rural Gabon, close contact between humans and livestock within family farming systems, coupled with inadequate hygiene, increases the risk of cross-species transmission of these parasites (Feng and Xiao, 2011). This underscores the need for integrated One Health approaches combining animal health management, public health education, and environmental sanitation in future surveillance and control efforts. Differences between univariate and multivariate model outcomes highlight the complexity of parasite-host-environment interactions. For example, while physiological status was significant in univariate analyses, it lost significance in multivariate models likely due to confounding by age, sex, or farming system variables. This emphasizes the importance of multivariate approaches to discern independent predictors and avoid misleading conclusions based on crude associations (Vaumourin et al., 2014). Such analyses enable a more precise identification of risk factors and prioritization of intervention targets.

Our results highlight that the prevalence and intensity of gastrointestinal parasites in small ruminants are not uniform across genera but exhibit distinct associations with various host and environmental factors, underscoring the need for genus- or group-specific approaches in parasite control. For instance, the genera *Eimeria* and the strongyles 2 showed heightened sensitivity to factors such as season and farming system. The increased prevalence of *Eimeria* spp. during the rainy season can be explained by the favorable climatic conditions, notably higher humidity and moderate temperatures, which enhance oocyst sporulation and survival on pastures (Al-Natour et al., 2002; Castro et al., 2023). Similarly, strongyles 1 and strongyles 2, both strongyles with free-living larval stages, are greatly influenced by environmental conditions that affect larval development and infective stage availability, making their transmission highly seasonal in tropical settings (Santos et al., 2012). Their resilience and adaptability to a wide range of conditions, combined with their hematophagic feeding behavior (strongyles 1), contribute to their dominance and clinical significance. Moreover, farming system notably influenced the prevalence of several genera, with extensive systems showing higher infection rates, likely due to increased exposure to contaminated grazing areas, poor sanitation, and lack of targeted anthelmintic treatments (Badran et al., 2012; Odoi et al., 2007). For example, *Strongyloides* and *Balantioides*, parasites with direct life cycles or resistant cyst stages, are particularly favored by such environmental conditions, raising concerns for both animal and human health given their zoonotic potential (Fleitas et al., 2022; White et al., 2019). These genus-specific patterns illustrate that blanket approaches to parasite control may not be optimal. Instead, tailored strategies considering the biology and epidemiology of each parasite genus, such as timing treatments to target peak transmission seasons of particular parasites or adjusting management practices to disrupt specific life cycles, are essential for more effective and sustainable control (Kenyon et al., 2009; Van Dijk and Morgan, 2012). Finally, the recognition of genus-specific risk factors also provides valuable insights for designing integrated surveillance systems and One Health interventions that address both animal health and zoonotic risks in rural Gabonese communities (Coyle et al., 2020).

Despite providing valuable insights into gastrointestinal parasitism in small ruminants in Franceville, several limitations must be acknowledged. First, the relatively small sample size, particularly the low number of goats included (*n* = 7), constrained the robustness of interspecies comparisons and reduced the statistical power for subgroup analyses. Increasing sample sizes for these underrepresented categories would enhance future investigations. Second, the absence of molecular diagnostic techniques and larval culture methods limited the ability to precisely differentiate morphologically similar parasite species, notably *Oesophagostomum* spp.*, Haemonchus contortus* and *Bunostomum* spp. This diagnostic limitation may have led to under- or over-estimation of certain genera and concealed species-specific epidemiological patterns critical for targeted control (Moniot et al., 2024). Third, potential sampling biases arose from the timing and conditions of sample collection. The study coincided with the post-Ramadan period, during which traditional animal slaughter practices reduced herd sizes and altered population structure on farms. This temporal window may not fully represent parasite prevalence and burden throughout the year, affecting the generalizability of results. Finally, the cross-sectional design inherently limits understanding of the temporal dynamics of parasite infections, such as seasonal variation in egg shedding or reinfection rates. The lack of longitudinal data precludes assessment of infection persistence, reinfection cycles, and the impact of repeated treatments, which are crucial for designing effective control programs (Castro et al., 2023). Addressing these limitations through expanded, longitudinal studies incorporating molecular diagnostics would substantially improve knowledge of the epidemiology of gastrointestinal parasites in Gabonese small ruminants.

This study highlights a critical need to improve sanitary management of small ruminants in Franceville, particularly through targeted interventions focusing on vulnerable groups such as pregnant females who exhibit heightened parasite burdens. Educational programs for farmers emphasizing hygienic practices, strategic deworming, and optimized husbandry are paramount to reducing parasite transmission and mitigating health impacts (Odoi et al., 2007). Continuous veterinary surveillance, including differentiated diagnostics attuned to seasonal fluctuations and distinct farming systems, is essential to monitor infection trends and guide timely interventions. Such adaptive surveillance would enable more precise parasite control, reduce economic losses, and limit the development of anthelmintic resistance (Moniot et al., 2024). Future research directions should prioritize the integration of molecular tools (e.g., PCR, qPCR) to refine species-level identification and ascertain parasite population structures and resistance profiles. Longitudinal studies tracking parasite dynamics over multiple seasons and treatment cycles would further elucidate infection patterns and intervention efficacy. Moreover, incorporating anthelmintic resistance monitoring is increasingly crucial given its global rise and impact on sustainable parasite control (D'Ambrosio et al., 2025). At a policy level, these findings underscore the necessity for tailored veterinary health programs that address parasitism in small ruminants as a key component of national food security and rural livelihoods in Gabon. Strengthening veterinary extension services and reinforcing regulatory frameworks on animal health could support the implementation of evidence-based parasite management strategies in Franceville and beyond.

## 5 Conclusion

This study has highlighted the significant prevalence and complexity of gastrointestinal parasitic infections in small ruminants in Franceville, Gabon, revealing a high overall prevalence (91.7 %) and a rich diversity of parasites, with moderate to severe infection intensities, particularly among nematodes and protozoa. Risk factor analysis underscored the pivotal roles of age, sex, physiological status, especially pregnancy, as well as husbandry practices and seasonal variations in shaping parasite dynamics. These findings emphasize the need for targeted, genus-specific approaches and integrated control strategies that consider the ecological and biological characteristics of the parasites, the predominance of extensive farming systems, and critical periods such as gestation. Methodological limitations, including the small sample size for goats and the absence of molecular diagnostics, highlight the necessity for further research incorporating longitudinal monitoring and genetic analyses to deepen epidemiological understanding and optimize interventions. Finally, the notable presence of zoonotic parasites calls for a One Health approach that integrates animal, human, and environmental health to enhance disease control and food safety in the region. These data provide a crucial foundation to guide veterinary policies and farmer support initiatives in Gabon.

## CRediT authorship contribution statement

**Krista Mapagha-Boundoukou:** Writing – review & editing, Writing – original draft, Methodology, Investigation, Formal analysis, Data curation. **Larson Boundenga:** Writing – review & editing, Writing – original draft, Validation, Supervision, Project administration, Methodology, Data curation, Conceptualization. **Mohamed H. Mohamed-Djawad:** Writing – review & editing, Formal analysis, Data curation. **Neil M. Longo-Pendy:** Software, Formal analysis, Data curation. **Patrice Makouloutou-Nzassi:** Writing – review & editing, Software, Methodology, Formal analysis, Data curation. **Mourad Ben Said:** Writing – review & editing, Validation, Methodology, Formal analysis, Data curation. **Gael Darren Maganga:** Writing – review & editing, Visualization, Supervision, Project administration, Methodology, Formal analysis, Data curation, Conceptualization.

## Consent for publication

Informed consent was obtained from the owners.

## Ethics approval and consent to participate

This study was approved by the Scientific Committee of our institute, the Centre Interdisciplinaire de Recherches Medicales de Franceville (CIRMF), in accordance with the ethical principles of animal research. All samples were taken with due regard for animal welfare, and during this study, all samples were collected with the consent of the animal owners. Furthermore, the procedures for animal sampling were evaluated by the Institutional Committee for Animal Use and Care of the National Higher Institute of Agronomy and Biotechnology (INSAB) at the University of Sciences and Technology of Masuku (USTM, Gabon).

## Funding

No funding was received for this study.

## Declaration of competing interest

The authors declare that the research was conducted in the absence of any commercial or financial relationships that could be construed as a potential conflict of interest.

## Data Availability

All data generated or analyzed during this study are included in this published article.

## Appendix A Supplementary data

Table S1. Moprhometric classification od strongyle-type eggs Image 1
